# Epidemiological characteristics of overseas imported COVID-19 cases into China: A scoping literature review

**DOI:** 10.3389/fpubh.2023.1143468

**Published:** 2023-03-29

**Authors:** Zitong Zhang, Yifeng Chen, Qingyu Li, Yan Yang, Jiake Chen, Yan Lin, Zhihong Xiao, Marie Ma, Chuancheng Wu, Baoying Liu, Rongxian Xu, Jianjun Xiang

**Affiliations:** ^1^School of Public Health, Fujian Medical University, Fuzhou, Fujian Province, China; ^2^Magill Medical Center, Adelaide, SA, Australia

**Keywords:** imported, COVID-19, epidemiological characteristics, review, China

## Abstract

Previous studies investigating the characteristics of imported cases were mostly limited to a certain province/city or a specific sub-group during a certain period with a small sample size, which may not provide an overall picture of the characteristics of imported cases. In this scoping literature review, we comprehensively synthesized the epidemiological characteristics of overseas imported COVID-19 cases into China by retrieving six literature databases, with aims to provide implications for more targeted control, prevention, and medical treatment of this disease. After dropping duplicates and reviewing titles, abstracts, and full-texts, 50 articles were included in the review finally, including 26 (52%) articles in English and 24 (48%) articles in Chinese. According to the type of data sources, the 50 studies were divided into three categories: 13 (26%) articles using data sourced from the Chinese Infectious Diseases Online Reporting System, 15 (30%) articles using data from the websites of national/local health departments, and 22 (44%) articles using hospital admission data. Most of the overseas imported COVID-19 cases were young and middle-aged Chinese students and businessmen returning from the United States, Europe, and some neighboring countries. Airport routine health screening measures could not identify COVID-cases effectively, although scheduled multiple nucleic acid tests were required before boarding. Almost all imported cases were identified during the hotel quarantine period. Although a large proportion of imported cases were asymptomatic or with mild symptoms in the published literature, they may be due to participant selection bias. The exact proportion of asymptomatic cases may need to be further investigated especially through population-based large-scale studies.

## 1. Introduction

**Figure 1:**
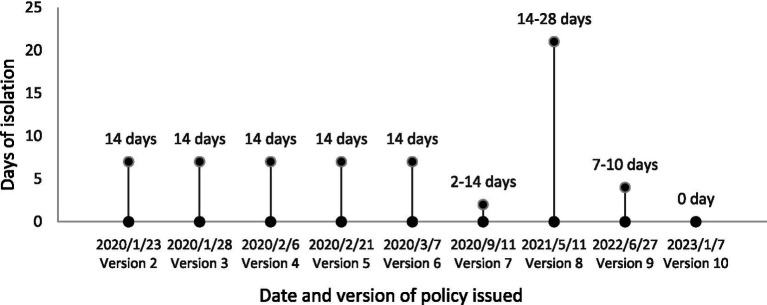
Duration of mandatory hotel/home quarantine for incoming passengers according to the 2nd–9th editions of the COVID-19 prevention and control guidelines.

Since the first case of COVID-19 was identified in late-December in Wuhan, Hubei Province of central China, the epidemic spread rapidly and was declared a global pandemic by WHO on 11 March 2020 (1). COVID-19 is one of the most widespread epidemics in human history, not only posing a huge threat to the health of vulnerable populations (e.g., older adults) but also severely impacting global economic development (2). Looking back at China’s tremendous efforts in fighting against COVID-19 in the past 3 years, the whole process could be roughly divided into four stages (3). The first stage is the formation of the COVID-19 epicenter in Wuhan and its spread to other provinces from 31st December 2019 to 29th February 2020; The second stage lasted from 1st to 21st March 2020, characterized with the containment of COVID-19 outbreaks and the number of cases was reduced to less than 10 in most provinces; The third stage is the sporadic outbreak mostly triggered by overseas imported cases from March 2021 to June 2022. In this stage, the priority of precautionary measures has gradually shifted from domestic infected cases to overseas imported cases. To prevent overseas imported cases, since 29th March 2022 Civil Aviation Administration of China introduced the “Five-One” policy to limit the number of international flights, namely each airline can only operate one flight per week to travel to and out of China (4). Moreover, a flight would be suspended for 1–2 weeks when confirmed cases accounted for a certain percentage (e.g., 4%) of inbound passengers. The duration of hotel/home quarantine for incoming passengers was updated correspondingly (Figure 1), according to the domestic and international COVID-19 epidemic situations. The fourth stage is the relaxation of strict COVID-19 restrictions (e.g., case tracing) since 7th December 2022. COVID-19 was initially classified as a B-category notifiable infectious disease in China but managed under A-category protocols. From 8th January 2023, control measures against COVID-19 have been downgraded from A-category to B-category (5).

The infectivity and virulence of SARS-CoV-2 evolve rapidly, and the corresponding prevention guidelines have been updated to the 10th edition in China (6). Under the new policy, the control strategy has shifted from the hard-line “zero-COVID” measures such as strict lockdown and large-scale all-staff COVID-19 testing to co-exist with the virus. The current priorities include the protection and medical treatment of infected vulnerable populations, increasing the vaccination rate of older adults, strengthening surveillance, and optimizing border control. International flights are anticipated to restore gradually. The duration of hotel quarantine has been reduced from at least 2–3 weeks in a certain period to 5 days in November 2022. From 8th January 2023, quarantine requirements on inbound travelers have been canceled. Overseas imported cases into China are likely to increase in the following several months because of the relaxed international travel restrictions (e.g., the cancelation of hotel quarantine), which may burden the already overloaded healthcare system, especially in the under-developed rural areas.

Most of previous studies focused on the spatial and temporal distribution of overseas imported COVID-19 cases, and they were published at an early stage with a small sample size (7, 8). In this scoping literature review, we aimed to comprehensively synthesize the epidemiological characteristics of overseas imported COVID-19 cases into China, to provide implications for more targeted control, prevention, and medical treatment of this disease.

## 2. Methods

### 2.1. Search strategy

**Figure 2:**
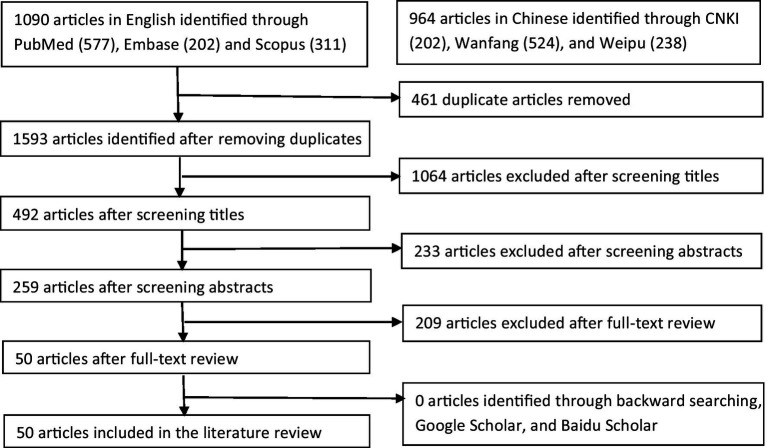
Flow chart of literature search process.

Articles involving epidemiological characteristics of overseas imported COVID-19 cases into China were searched using the combination of keywords: imported COVID-19 AND (China OR mainland China OR Taiwan OR Hongkong OR Macao), including studies published from the time of database creation to November 10, 2022. Literature databases used for this review included PubMed, Embase, and Scopus. Three Chinese literature databases, China National Knowledge Infrastructure (CNKI), Wanfang, and Weipu, were also retrieved to avoid language bias, as the Ministry of Science and Technology of China released a notice on 29 January 2020 to encourage Chinese researchers to publish COVID-19 related studies in domestic journals (9). The initial search results were imported into an Endnote library. Duplicate records were removed using the EndNote function of “find duplicates.” Appropriate peer-reviewed studies were identified by a three-step process (Figure 2): screening titles, reviewing abstracts of articles that were difficult to judge by screening their titles, and reviewing the full-texts. Studies were independently appraised by two investigators (ZZH and JX). Where consensus could not be reached, there was a group discussion to determine the final articles included for reviewing. Reference lists and similar articles recommended by PubMed were also scanned for additional articles not previously identified. The ‘Google Scholar’ and ‘Baidu Scholar’ search engines were also used to retrieve relevant literature.

This scoping review of overseas imported cases includes articles from three data sources: China Infectious Disease Reporting System, national/local health department websites, and hospital inpatient data. Due to the different emphases of the source data, we divided the collected literature data into three tables to facilitate the statistics of useful information.

### 2.2. Inclusion and exclusion criteria

The studies selected in this review met the following criteria:

- Investigated the epidemiological characteristics of overseas imported COVID-19 cased into China.
- Peer-reviewed studies published from database inception to 10 November 2022.
- Conference abstracts, letters, editorials, field investigations, reports and unrefereed preprints on medRxiv and bioRxiv were excluded.

## 3. Results

Figure 1 shows the process of selection of articles for inclusion in the review. An initial search generated 2054 articles, with 53.1% being published in English and the rest in Chinese. After dropping duplicates and reviewing titles, abstracts, and full-texts, 50 articles were finally included in the review, including 26 (52%) articles in English and 24 (48%) articles in Chinese. According to the type of data sources, the 50 studies were divided into three categories: 13 (26%) articles using data sourced from the Chinese Infectious Diseases Online Reporting System, 15 (30%) articles using data from the websites of national/local health departments, and 22 (44%) articles using hospital admission data. They were summarized in Tables 1–3, respectively.

**Table 1:** Summary of 13 studies using data sourced from the Chinese Infectious Diseases Reporting System.

Author	Data period	Number of imported cases	Number of source countries and top 3	Age	Gender (% of males)	Reason for traveling (top3)	Chinese nationality (%)	Days from arrival to diagnosis	Asymptomatic case (%)	Clinical classification (Mild: Moderate: Severe: Critically)	Cases infected by imported	Identified through airport screening (%)	Province/municipality of entry
Fang et al. (10).	5 Mar. to 30 Mar. 2020	171	24 and United Kingdom (37.3%), United States (18.6%), and France (11%)	Median: 23 years	56.7	Study (56.6%)	71.3	2.9	0.6	69:96:4:1	–	18.7	Shanghai
						Work (24.6%)							
				18–40 years: (77.7%)		Business (5.3%)							
Zhen et al. (11)^.^	3 Mar. to 1 Apr. 2020	103	22 and United Kingdom (26.2%), Philippine (12.6%), and United States (12.6%)	Median: 31 years	61.2	Business/work (39.8%)	75.7	3.0	14.1	38:54:0:0	36	47.6	Guangzhou
						Study (35.0%)							
				Range: 11–63 years		Unemployed (12.6%)							
Liu et al. (12).	1 Mar. to 7 Apr. 2020	91	19 and Italy (25.3%), United Kingdom (18.7%), and Spain (17.6%)	Mean: 33.7 years	52.8	Study (34.1%)	86.8	3.0	47.3	16:32:0:0	–	–	Zhejiang
				Range: 7–73 years		Business/work (23.1%)							
Dong et al. (13).	1 Jun.to 30 Sep.2021	171	23 and Philippines (24%), U.A.E (22.8%), and United Kingdom (14.6%)	Median: 28 years	66.7	Work (33.3%)	80.1	More than 14 days: 10. %	77.8	132:37:1:1	–	13.5	Beijing
				Range: 10 ms–64 years		Study (24.6%)							
						Unemployed (18.71%)							
Zhao et al. (14).	Up to 18 Jun.2021	207	Africa	18–40 years (72.9%)	69.6	Business (62.3%)	26.1	4.0	87.0	5:22:0:0	66	0.0	Guangzhou
						Study (18.4%)							
						Unemployed (15%)							
Yu et al. (15).	15 Mar.2020 to 31Aug. 2021	552	53 and Spain (14.1%), France (13.2%), and United States (13.0%)	20–40 years (59.8%)	63.9	Unemployed (18.8%)	91.8	–	44.2	106:139:0:0	–	45.5	Tianjin
				Range: 3-77 years		Business (14.7%)							
Chen et al. (16).	29 Jan. to 12 Jul. 2020	72	18 and United States (22.2%), Russia (20.8%), and United Kingdom (15.3%)	Mean: 35.9 years	51.5	Study (43.1%)	87.5	–	54.2	5:28:0:0	0	–	Liaoning
				Range: 14–74 years		Business/work (13.9%)							
Chen et al. (17).	19 Mar.2020 to 31 May.2021	325	44 and Philippines (19.7%), United States (15.4%), and Russia (8.9%)	Median: 36.1 years	71.0	Work (25.2%)	–	1.5	0	76:249:0:0	0	40.9	Fujian
				Range: 1 ms–71 years		Business (15.4%)							
						Study (15.1%)							
Li et al. (18).	1 Jan.2020 to 31 Jul.2021	77	15 and Russian (19.5%), Japan (19.5%), and Philippines (13%)	21-40 years: (59.7%)	90.1	Fisherman (66.2%)	–	–	58.4	1:10:0:0	–	–	Dalian
						Work (6.5%)							
						Study (5.6%)							
Qi et al. (19).	1 Sept. 2020 to 28 Jan. 2021	136	32 and Philippines (19.1%), India (10.3%), and Nigeria (8.8%)	Mean: 34.9 years	67.7	Business/work (33.1%)	–	–	72.1	7:30:1:0	–	–	Zhejiang
				Range: 6 ms -6 years									
Hu et al. (20).	28 Feb. to 30 Nov. 2020	450	9 and Iraq (8.4%), Egypt (4.0%), and Ethiopia (4.0%)	Median: 34 years	82.9	Business/work (40.9%)	87.3	–	40.9	54:212:0:0	193	73.9	Chengdu
				Range: 2 ms -70 years									
Zhang et al. (21).	11 Mar. to 6 Jul. 2020	268	32 and United Kingdom (20.1%), Bangladesh (16.8%), and United States (15.3%)	Median: 32 years	59.0	Study (34.9%)	89.2	–	31.7	–	36	73.5	Guangzhou
				Range: 3–70years		Business/work (32.6%)							
Liu et al. (22).	21 Jan. to 6 Apr. 2020	321	37 and United States (25.2%), United Kingdom (22.7%), and France (6.5%)	20–39 years: (61.1%)	47.0	Tourism (32.4%)	96.6	6.3	3.4	–	52/16	32.7	Taiwan
						Business/work (27.4%)							
						Study (26.5%)							

**Table 2:** Summary of 15 studies using data sourced from the websites of national/local health departments.

Author	Data period	Number of imported cases	Number of source countries and top 3	Age	Gender (% of males)	Reason for traveling (%)	Chinese nationality (%)	Days from arrival to diagnosis	Asymptomatic case (%)	Cases infected by imported	Identified through airport screening (%)	Province of entry
Lam et al. (23).	1 Jan. to 31 May 2020	657	NA and United Kingdom (61.6%), United States (13.4%), and France (7.2%)	15–24 years: (40%)	28.7	Study (37.3%)	–	2.6	20.8	250	25.6	Hong Kong
Chen et al. (24).	1 Mar. to 2 Jun.2020	200	31 and United Kingdom (28.5%), United States (17.5%), and Philippines (8.5%)	Median: 31.6 years	63	Living abroad (11.5%)	81	4.3	79.5	–	95.0	Guangdong
				Range: 2–70 years								
Wang et al. (25).	13 Feb.2021	31	1 and Kenya	Mean: 45.8 years	96.8	Work (74.2%)	97.2	–	20.0	–	100.0	Guangdong
				Range: 27–61 years								
Yang et al. (26).	1 Jan. 2020 to 28 Feb.2021	1,585	9 and Philippines (48.9%), Indonesia (19%), and Singapore (17.6%)	Median: 33 years	79.8	Work (21.3%)	–	Median1 Range0-27	58.1	–	–	Mainland China
				Mean: 35.2 years		Unemployed (13.6%)						
				Range: 1–70 years		Business (12.9%)						
Li (27).	24 Mar. to 15 Sep.2020	184	-	Median: 32 years	54.5	Study (36.41%)	–	–	0.0	0	–	Inner Mongolia
				Range: 13–72 years		Business (45.7%)						
						Unemployed (4.3%)						
Cao (28).	5 Mar. to 31 Dec.2020	90	9 and Iran (41.1%), Saudi Arabia (22.2%), and Russia (12.2%)	Mean: 27.4 years	78.9	Study (53.3%)	100%	–	25.6	–	–	Gansu
				Range: 4 ms to 62 years		Work (11.1%)						
						Unemployed (8.9%)						
Tian et al. (29).	18 Mar.2020	5	1 and United Kingdom	Median: 38 years	40	Visit	–	1.8	20.0	1	40.0	Beijing
				Range: 1–69 years								
Ma et al. (30).	24 Mar. to 16 May.2020	19	5 and Russia (57.9%), United States (21.0%), and Thailand (10.5%)	–	6/10	–	–	–	–	–	–	Jilin
Shen et al. (31).	27 Feb. to 15 Aug. 2020	2,278	66 and Russia (33.2%), United Kingdom (14.4%), and United States (10.1%)	Range: 2 ms to 76 years	65.0	–	–	–	7.7	–	–	Mainland China
Chen et al. (32).	1 Mar. to 10 Apr. 2020	179	28 and United Kingdom (29.6%), United States (17.9%), and Philippines (9.5%)	Mean: 31.6 years	62.0	–	78.8	4.2	81.0	–	91.6	Guangdong
				Range: 1–70 years								
Li et al. (33).	26 Feb. to 18 Mar. 2020	188	18 and Iran (25.0%), Italy (22.3%), and Spain (15.4%)	Mean: 33.5 years	57.5	Business/work (45.4%)	94.2	–	0.5	80	–	Mainland China
				Range: 1–70 years		Study (25.9%)						
Li et al. (34).	23 Jan. to 8 Aug.2020	1,074	-	15–24 years most	–	Study/tourism	–	–	–	2,198	–	Hong Kong
Yang et al. (35).	1 Apr. to 31 Jul.2020	187	8 and Philippines (45%), United Kingdom (35%), and United States (14%)	–	–	–	–	–	–	–	–	Hong Kong
Zhao et al. (36).	1 Jan. to 19 Feb. 2020	45	-	20–59 years: (80.4%)	57.7	–	–	–	0.2	–	–	Jilin
Guo et al. (37)^.^	29 Feb. to 20 May 2020	1,709	50 and Russia (40.1%), United Kingdom (18.0%), and United States (10.7%)	Mean: 35.4 years	58.9	Study (40.0%)	–	–	–	–	–	Mainland China
				Range: 2 ms to 72 years								

**Table 3:** Summary of 22 studies using hospital admission data.

Author	Data period	Number of imported cases	Number of source countries and top 3	Age	Gender (% of males)	Reason for traveling (%)	Days from arrival to diagnosis	Asymptomatic case (%)	Clinical classification (Mild: Moderate: Severe: Critically)	Clinical symptoms (top 2)	Comorbidities (top 2)	Province of entry
Chen et al. (38)	25 Jan. to 20 Feb.2020	29	–	Median: 39 years	69	–	2.4	3.4	–	Fever (17.6%)	-	Chongqing
										Cough (12.5%)		
Liu et al. (39)	5 Mar. to 22 Mar.2020	58	9 and United Kingdom (32.8%), Italy (15.5%), and United States (15.5%)	Median: 29 years	53.4	–	3	8.6	–	Fever (50.0%)	Hypertension (12.1%)	Shanghai
										Cough (41.4%)	Diabetes (6.9%)	
Zhai et al. (40).	15 Mar. to 30 Apr.2020	53	6 and United Kingdom (49%)	Median: 27 years	39.6	–	–	0.0	4:6:0:0	Cough (52.8%)	In total (13.2%)	Beijing
				Moderate: 23 years						Sore throat (50.9%)		
Zhang et al. (41).	29 Mar. to 31 Aug.2020	79	10 and Singapore (34.18%), Russia (22.78%), and Kazakhstan (11.46%)	Mean: 38 years	88.7	Business/work mostly	6	24.2	2:4:1:0	Cough (22.8%)	Hypertension (67.6%)	Xi’an
				Range: 19–57 years						Fever (11.4%)	Diabetes (2.5%)	
Liu et al. (42).	29 Feb. to 27 Mar.2020	109	–	Median: 24 years	58.7	Work/study mostly	1	–	44:62:3:0	Fever (39.1%)	Diabetes (1.8%)	Beijing
				Mean: 27.3 years						Cough (33.3%)	Hypertension (2.8%)	
Hu et al. (43).	1 Jul.2020 to 15 Jan. 2021	23	–	Mean: 45.1	56.5	–	–	–	Severe:0	Fever (87%)	-	Hubei
				Range: 23–72 years						Cough (65.2%)		
Yong et al. (44).	1 Dec.2020 to 15 Ap.2021	75	7 and Nigeria (38.6%), Egypt (38.7%), and Ethiopia (16%)	–	90.7	–	–	20.0	53:7:0:0	Cough (9.3%)	-	Sichuan
										Sore throat (6.7%)		
Du et al. (45).	23 Jan. to 19 Feb. 2020	33	–	30–59 years (69.7%)	60.6	–	4	0.0	–	Fever (78.8%)	-	Inner Mongolia
										Cough (48.5%)		
Li et al. (46).	29 Feb. to 20 Mar.2020	71	11 and Spain (31.0%), United Kingdom (23.9%), and Italy (22.5%)	Median: 24 years	38.0	–	4	2.8	–	Cough (49.3%)		Beijing
										Fever (42.3%)		
Li et al. (47).	22 Mar. to 17 May.2021	46	8 and Pakistan (39.1%), Sudan (21.7%), and United Arab Emirates (19.6%)	Mean: 40.5 years	89.1	–	–	67.4	7:3:0:0	Fever (6.6%)	Hypertension (13%)	Shanxi
										Cough (6.6%)	Diabetes (6.6%)	
Yan et al. (48).	28 Jul.2020 to 31 Dec.2021	137	22 and Uzbekistan (20.4%), Singapore (12.4%), and Germany (10.9%)	Median: 38 years	89.8	–	<3d 67.8% >3d 32.2%	48.9	5:5:0:0	Fever (22.6%)	Hypertension (7.3%)	Xian
				Mean: 37.8 years						Cough (18.9%)	Diabetes (2.2%)	
				Range: 8–68 years								
Liu et al. (49).	16 Dec. to 31 Dec. 2021	17	–	30-40 years: (52.9%)	94.1	–	–	11.8	2:1:0:0	Sore throat (58.8%)	In total 17%	Hunan
										Cough (47%)		
Qiu et al. (50).	19 Mar. to 1 Sept. 2020	10	10 and Italy (50.0%), Spain (30.0%)	Median: 45 years	30.0	–	4.0	20.0	1:9:0:0	Cough (60%)	In total 20%	Zhejiang
				Range: 18–73 years						Fever (50%)		
Cai et al. (51).	14 Mar. to 8 Apr. 2020	38	10 and United Kingdom (26.3%), Italy (13.2%), and France (10.5%)	Median: 14.5 years	57.9	Study 91.4%	–	42.1	8:14:0:0	Cough (28.9%)	–	Shanghai
				Range: 2.3–17 years						Fever (18.4%)		
Chen et al. (52).	1 Feb. to 31 Mar. 2020	90	na and Africa (20.0%), Europe (4.4%)	Mean: 38.7 years	64.4	–	3.2	31.1	28:52:2:0	Cough (40.0%)	Diabetes (5.6%)	Guangdong
										Fever (31.0%)	Hypertension (5.6%)	
Luo et al. (53).	20 Jan. to 31 Oct. 2020	78	Mainly United Kingdom, Philippines, and United States	Mean: 35 years	76.9	Study mostly	1	50.0	39:38:1:0	Cough (16.7%)	Hypertension (5.1%)	Fujian
				14–40 years: (66.7%)						Sore throat (11.5%)	Diabetes (1.3%)	
Zhang et al. (54).	20 Jan. to 20 Mar. 2020	69	11 and United Kingdom (26.1%), Spain (24.6%), and Italy (24.6%)	Median: 27 years	40.6	Study 44.9%	4	2.9	29:37:2:1	Cough (43.5%)	In total 21.7%	Beijing
				Range: 6–69 years		Business/work 17.4%				Fever (39.1%)		
Dan et al. (55).	Oct.2020 to May 2021	177	na and United States (11.11%), Zambia (6.84%), and Nigeria (6.84%)	Median: 34 years	76.8	–	1.7	–	24.3	–	–	Guangdong
				Range: 16–85 years								
Yuan et al. (56).	14 Mar. to 3 Apr. 2020	41	5 and United Kingdom (68.3%), United States (14.6%), and Spain (7.31%)	Mean: 27.4 years	31.7	–	–	–	24:17:0:0	Cough (31.7%)	0	Beijing
										Fever (26.8%)		
Zhao et al. (57).	21 Jan. to 5 Apr. 2020	7	3 and Russia (42.9%), United Kingdom (42.9%), and Denmark (14.3%)	Mean: 23.6 years	71.4	–	–	71.4	0:6:1:0	Fever (14.3%)	In total 28.6%	Hebei
										Diarrhea (14.3%)		
Bi et al. (58).	19 Mar. to 24 Apr.2020	56	8 and United Kingdom (32.1%), United States (26.8%), and France (19.6%)	Mean: 29.3 years	50	–	–	0.0	0:51:3:2	Cough (42.9%)	Hepatitis B (8.9%)	Tianjin
										Fever (37.5%)	Diabetes (7.1%)	
Bao et al. (59).	23 Jan. to 3 Sep.2020	79	–	Mean: 28 years	74.7	Work/study mostly	–	1.3	37:39:2:0	Cough (24.1%)	Hypertension (3.8%)	Gansu
										Fever (16.5%)	Coronary heart disease (1.3%)	

Table 1 summarized the key characteristics of 13 articles analyzing imported cases reported through the online Chinese Infectious Diseases Reporting System (CIDRS). The number of source countries ranged from 5 to 53 and the most frequent top-three countries were United States, United Kingdom, and Philippines. The ranges of median and mean ages were 23–36 and 34–36 years, respectively. Most imported cases were males, with the proportion reaching as high as 90.1%. Study, work, and business were the most frequent reasons for traveling. The vast majority of cases were Chinese citizens (71.3–96.6%). The number of days from entry to diagnosis ranged from 1.5 to 6.3, while it should be noted that there was a special case taking 14 days to be diagnosed. The proportion of asymptomatic cases varied largely from zero to 87%. The proportion of cases identified through airport health screening measures ranged from zero to 74%.

Table 2 showed the characteristics of 15 articles analyzing COVID-19 cases sourced from the websites of national or local health departments. Basically, the characteristics of imported cases in Table 2 were similar with that in Table 1 in the aspects of reasons for traveling and days from entry to diagnosis, because COVID-19 data released on the official websites were also sourced from the CIDRS. By contrast, characteristics of imported cases based on hospital admission data (Table 3) were slightly different with cases sourced from the CIDRSA and governmental websites in Tables 1, 2. The number of source countries reached 177 by May 2021. Moreover, they were relatively more diverse compared to that in Tables 1, 2. The proportion of asymptomatic cases ranged from zero to 71.4%, which was lower than that in Tables 1, 2. The most frequent clinical symptoms of imported cases were fever, coughing, and sore throat, with the highest percentages reaching 87, 65, and 51%, respectively. The most common comorbidities were hypertension and diabetes.

## 4. Discussion

Imported COVID-19 cases are an important source triggering local sporadic outbreaks. One imported case could reportedly result in more than 2,000 infections in a short period of time. Thus, strict border control measures had been taken by the government to reduce the risk, such as scheduled multiple nucleic acid tests before boarding, a “Five-One” flight policy, closed-loop management, and hotel quarantine. Previous studies investigating the characteristics of imported cases were mostly limited to a certain province/city or a specific sub-group during a certain period, which may not provide an overall picture of the characteristics of imported cases. In this study, we comprehensively reviewed the epidemiological characteristics of overseas imported COVID-19 cases into China, retrieving six literature databases. Findings of this literature review may not only provide evidence for the development of current control measures against COVID-19 but also facilitate the management of imported cases.

We found that the importing countries were mainly high-income countries (e.g., United States and United Kingdom) or neighboring countries (e.g., Russia and Malaysia) with close trade links with China. United States and United Kingdom are the top two destinations for Chinese students. In 2021, many overseas Chinese students were selected to return to China due to the following reasons. First, many western countries abandoned case tracking, early detection, and case isolation and selected coexistence with the virus, leading to the surge of COVID-19 related morbidity and mortality (60). By contrast, China took a strict zero-case policy at that time and the epidemic was well-contained. Second, some universities transferred to online teaching temporarily to avoid campus outbreaks. Third, evidence has shown that the well-being of Chinese international students deteriorated in the early stage of COVID-19 pandemic. Over the debate of COVID-19 origin, a high prevalence of mental health issues (e.g., depression, anxiety, and feeling of discrimination) was observed among them (61, 62). In addition, work and business activities were also the most common reasons for traveling to China, while most incoming travelers were Chinese nationals. International air flights are the major way for imported cases (63), however, some travelers sought to enter *via* land or port when most international flights were suspended at the early stage of the global pandemic. For example, most cases imported from Russia were through land border ports, especially the Suifen River Estuary in Mudanjiang city. The proportion of male travelers was higher than their female counterparts. Our result is supported by the findings of a global study of population mobility networks (61), which utilized the characteristics of travelers and geographical factors to predict the COVID-19 cross-border transmission.

According to the level of severity, COVID-19 was initially divided into four types: mild, moderate, severe, and critical cases. However, published literature shows that many infections of COVID-19 are asymptomatic (64). It has been reported that viral loads of asymptomatic patients were similar with those of symptomatic individuals (65), suggesting that asymptomatic patients have a similar capacity in infectivity for transmission. The potential transmission of asymptomatic infections poses a significant challenge to the control and prevention of COVID-19. Two prerequisites must be met for the diagnosis of asymptomatic COVID-19 infection: the absence of self-perceived or clinically recognizable symptoms; and a positive reverse transcription-PCR (RT-PCR) test. In this review, the proportion of asymptomatic cases was highly variable with a range from 0 to 87%. Not only in the imported cases, a highly varied proportion of asymptomatic infections was also reported in the general population (8). This may be due to participant selection bias. For example, in this review we found the average proportion of asymptomatic infections based on hospital admission data (22%) was lower than the proportion based on data sourced from CDC (41%) and the websites of local health departments (26%). Most of the imported cases were young and middle-aged who were less likely to have clinical manifestations than the vulnerable sub-groups (e.g., children, pregnant women, and older adults with chronic diseases). Moreover, symptomatic cases may opt to postpone their trips. Relative fewer cases from low- and middle-income countries due to the soaring airfares and the limited number of flights may also contribute to the participant selection bias. Another factor associated with clinical manifestations was the course of disease. Evidence has shown that the proportion of asymptomatic infections ranged from approximately 20–75% at initial testing, however, only 4% remained asymptomatic throughout the disease finally (66). In addition, vaccination status, the type of vaccines, and virus strain also affect the presentation of clinical symptoms. The exact proportion of asymptomatic cases needs to be further investigated especially through population-based large-scale studies.

We observed a high heterogeneity in sample size, patients’ age, COVID-19 symptoms, and comorbidities, although most of the included studies were conducted in China. Fever and coughing were the most common symptoms. Regarding comorbidities for patients with COVID-19, the highest severity factors were hypertension, diabetes, obesity, chronic obstructive pulmonary disease, and cardiovascular disease. This is consistent with a recent literature review focused on the general population (8). Pustahija et al. found there were no significant differences between travel-associated cases and cases identified in the general population in terms of the epidemiological and clinical characteristics in Serbia (67). Another study from Bolivia found similar epidemiological characteristics of imported COVID-19 cases with this study (68). It should be noted that the differences between studies in symptoms and comorbidities cannot be compared directly without taking demographical factors, virus strain, healthcare systems, selection criteria, the course of disease, and border control measures into account.

In response to the COVID-19 pandemic, many countries have imposed international travel restrictions to prevent the importation of COVID-19 cases. In this review, we found the proportion of imported cases identified by airport health screening measures varied considerably from zero to 100% with an average of 49.9%, indicating that entry screening alone may not detect imported cases effectively at borders. Although more than 90% of COVID-19 patients had a fever, body temperature might not be an adequate screening method as it may miss travelers in the incubation period or travelers concealing fever during travel (69). Despite the ineffectiveness of entry screening measures, travel restrictions may delay the transmission of COVID-19 between countries and have concomitant positive effects such as discoursing the travel of ill persons and raising the awareness of infectious disease control (63, 70). In this review, the number of days from entry to diagnosis ranged from 1.5 to 6.3 days. To prevent the importation of COVID-19 cases, 1–3 weeks’ hotel quarantine was mandatorily required by the Chinese government for all incoming passengers before 8th January 2023. Moreover, multiple RT-PCT tests were required before boarding and during the quarantine period to minimize the risk of importation as much as possible. However, it should be noted that quarantine hotels are not designed with infection control and there is a risk of within hotel transmission between guests and/or staff. It has been estimated that 8–11 per 1,000 cases identified during hotel quarantine may be infected by another unlinked case during quarantine (71). Therefore, its impact on the characteristics of imported cases should be minimal.

To maintain international personnel and economic exchanges, quarantine strategies have been adjusted timely according to the infectivity, virulence, and incubation period of different variants. A recent systematic review suggested that the incubation periods of Alpha, Beta, Delta, and Omicron variants were 5.0, 4.5, 4.4, and 3.4 days, respectively (72). With the shortening of the incubation periods of new variants, the quarantine period has been reduced accordingly. Now hotel quarantine requirements on inbound travelers are no longer required. Nevertheless, negative RT-PCR test 48 h before departure and online self-declaration of health status are still in place to prevent importation. These targeted measures significantly reduced the importation of COVID-10 cases into China and the pressure on the healthcare system (73). China will keep monitoring the characteristics of imported cases to adjust prevention policies to lessen the impact on economic and social development.

## 5. Conclusion

Most of the overseas imported COVID-19 cases were young and middle-aged Chinese students and businessmen returning from the United States, Europe, and some neighboring countries. Airport routine health screening measures could not identify COVID-cases effectively although scheduled multiple nucleic acid tests were required before boarding. Almost all imported cases were identified during the hotel quarantine period. Although a large proportion of imported cases were asymptomatic or with mild symptoms in the published literature, they may be due to participant selection bias. The exact proportion of asymptomatic cases needs to be further investigated especially through population-based large-scale studies.

## Author contributions

JX, RX, BL, and ZZ conceived the review. ZZ, JX, YC, QL, YY, JC, YL, and ZX participated in the literature search and selection. ZZ and JX drafted the manuscript and made the tables. ZZ, MM, CW, BL, RX, and JX revised the manuscript. All authors contributed to the article and approved the submitted version.

## Funding

This study was supported by the 2019 Minjiang Scholar Start-up Research Fund of Fujian Province.

## Conflict of interest

The authors declare that the research was conducted in the absence of any commercial or financial relationships that could be construed as a potential conflict of interest.

## Publisher’s note

All claims expressed in this article are solely those of the authors and do not necessarily represent those of their affiliated organizations, or those of the publisher, the editors and the reviewers. Any product that may be evaluated in this article, or claim that may be made by its manufacturer, is not guaranteed or endorsed by the publisher.
